# Functional assays provide a robust tool for the clinical annotation of genetic variants of uncertain significance

**DOI:** 10.1038/npjgenmed.2016.1

**Published:** 2016-03-02

**Authors:** Nicholas T Woods, Rebekah Baskin, Volha Golubeva, Ankita Jhuraney, Giuliana De-Gregoriis, Tereza Vaclova, David E Goldgar, Fergus J Couch, Marcelo Alex Carvalho, Edwin S Iversen, Alvaro NA Monteiro

**Affiliations:** 1 Eppley Institute for Research in Cancer and Allied Diseases, Fred & Pamela Buffett Cancer Center, University of Nebraska Medical Center, Omaha, NE, USA; 2 Cancer Epidemiology Program, H. Lee Moffitt Cancer Center, Tampa, FL, USA; 3 Cancer Biology PhD Program, University of South Florida, Tampa, FL, USA; 4 Instituto Nacional de Câncer, Programa de Farmacologia, Rio de Janeiro Brazil; 5 Human Cancer Genetics Programme, Spanish National Cancer Research Centre CNIO, Madrid, Spain; 6 Huntsman Cancer Institute and Department of Dermatology, University of Utah, Salt Lake City, Utah, USA; 7 Division of Experimental Pathology and Laboratory Medicine, Department of Laboratory Medicine and Pathology, Mayo Clinic, Rochester, MN, USA; 8 Instituto Federal do Rio de Janeiro, Rio de Janeiro, RJ, Brazil; 9 Department of Statistical Science, Duke University, Durham, NC, USA

## Abstract

Variants of Uncertain Significance (VUS) are genetic variants whose association with a disease phenotype has not been established. They are a common finding in sequencing-based genetic tests and pose a significant clinical challenge. The objective of this study was to assess the use of functional data to classify variants according to pathogenicity. We conduct functional analysis of a large set of *BRCA1* VUS combining a validated functional assay with VarCall, a Bayesian hierarchical model to estimate the likelihood of pathogenicity given the functional data. The results from the functional assays were incorporated into a joint analysis of 214 *BRCA1* VUS to predict their likelihood of pathogenicity (breast cancer). We show that applying the VarCall model (1.0 sensitivity; lower bound of 95% confidence interval (CI)=0.75 and 1.0 specificity; lower bound of 95% CI=0.83) to the current set of *BRCA1* variants, use of the functional data would significantly reduce the number of VUS associated with the C-terminal region of the BRCA1 protein by ~87%. We extend this work developing yeast-based functional assays for two other genes coding for BRCT domain containing proteins, *MCPH1* and *MDC1*. Analysis of missense variants in *MCPH1* and *MDC1* shows that structural inference based on the *BRCA1* data set can aid in prioritising variants for further analysis. Taken together our results indicate that systematic functional assays can provide a robust tool to aid in clinical annotation of VUS. We propose that well-validated functional assays could be used for clinical annotation even in the absence of additional sources of evidence.

## Introduction

Precision medicine approaches are based on the identification of molecular targets in the tumour or the host that can be used to identify at-risk individuals and inform treatment decisions resulting in improved outcomes. Large initiatives focused on identifying DNA alterations linked to disease risk in germline DNA, and to cancer initiation and progression in somatic (tumour) tissue DNA have offered tantalising evidence that the goal of personalised medicine can be achieved in the near future. However, the scale of data available exposes the challenge of how to annotate the numerous variants of uncertain significance (VUS) and distinguish high-risk from non-high-risk alleles (in germline DNA), and drivers from passengers (in tumour DNA). VUS are DNA alterations for which there is incomplete information about its disease association and the impact on the gene/protein function cannot be directly inferred. Traditionally, newly discovered germline variants suspected of being pathogenic are assessed by tests applicable to all genes such as segregation analysis, family history, population frequency, loss of heterozygosity analysis and gene-specific tests such as the presence of a microsatellite instability phenotype in tumours. This labour-intensive work is further hampered by low minor-allele frequency in these susceptibility gene alleles.^1^

It is clear that genome-wide discovery of germline and somatic VUS has far outpaced annotation, and there is a pressing need to provide scientifically rigorous alternatives for clinical annotation that can match data output.^2,3^ The development of computational prediction tools has been a focus of intense research. Direct assessment of variants using high-throughput functional assays can help with classifying variants and will be instrumental to benchmark the prediction models. To fill this gap, we propose that validated functional assays that interrogate individual alleles for specific molecular functions provide a robust tool for clinical annotation, especially for variants for which no other information may exist. As a proof of principle, we conducted an analysis of a large set of missense variants in the breast and ovarian cancer susceptibility gene *BRCA1*. Women who inherit inactivating mutations in *BRCA1* are at a significantly increased risk of developing early-onset breast and ovarian cancers.^4^ Classification of *BRCA1* variants as pathogenic or not pathogenic have implications for increased surveillance, prophylactic surgery and increasingly to inform therapy.

The study presented here completes the functional testing of all known missense variants in the C-terminal region of the BRCA1 protein using transcriptional assays^5^ and provides an extensive analysis of these variants using VarCall, a computational tool to predict the likelihood of pathogenicity^6^ given the results from functional assays. Finally, it has also been proposed that information from paralogous proteins could be used to identify potentially disease-causing variants.^7^ Here we apply this notion to protein modular domain families and test whether variants in the BRCT domains, a modular domain critical for signalling in the DNA damage response, of the tumour suppressor proteins MCPH1 and MDC1 can be predicted by structural inference from the larger set of *BRCA1* variants localised to the BRCT domains.

## Results

### BRCA1 variants in this study

**Figure 1:**
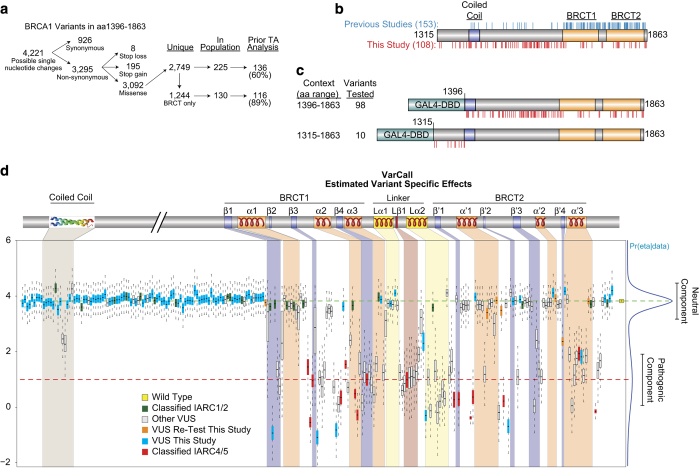
BRCA1 variant mapping. (**a**) Overview of *BRCA1* variants from amino acid residues 1396–1863. (**b**) Depiction of BRCA1 protein domains and motifs in the aa 1,315–1,863 region and the variants that have been tested by transcriptional assays in previous (blue) or the current (red) studies (**c**) Depiction of *BRCA1* constructs used in this study and the locations of the variants tested. (**d**) VarCall analysis of missense variants in the carboxy-terminal region (aa 1,315–1,863 of the BRCA1 protein). Transcriptional assays were performed for 98 missense variants in the aa 1,395–1,864 context (9 of these represent retests), 10 missense variants were tested by transcriptional assays in the aa 1,315–1,863 context (3 variants found in the population, 7 variants not currently known to occur in the population). Transcriptional assays were performed using a luciferase reporter system where 293T cells were co-transfected with a reporter plasmid, pG5Luc, which contains a firefly luciferase gene under the control of five GAL4 binding sites, a pCDNA3 plasmid coding for either wild-type or variant *BRCA1* constructs fused to the GAL4 DNA-binding domain, and an internal control containing a Renilla luciferase gene constitutively expressed (described in Carvalho *et al.* (2009))^44^. Results of these transcriptional assays were analysed using VarCall^6^ to predict the likelihood of pathogenicity. In addition, the coiled-coil domain and secondary structures of the BRCT domain were overlaid on the results. See Supplementary Figure S1 for additional details.

In human *BRCA1*, exons 13–24 encode a region from amino-acid (aa) residues 1,396–1,863 that can be used in transcriptional activation (TA) assays to determine the functional impact of missense variants.^8,9^ Each batch of assays was run with a positive (wild type) and negative (M1775R) control and each variant was tested in triplicate in at least two independent experiments (Supplementary Table S1). The variants tested here represent all known 89 variants for this region of *BRCA1* not previously analysed using the TA assays (Figure 1a; Supplementary Table S2). Nine variants were included for retesting from previous analyses (see Materials and Methods). We also tested 10 variants in a construct covering exons 11–24 (aa 1,315–1,863) to assess the function of the coiled-coil motif (CC; aa 1,392–1,424) and the preceding region (Figure 1b,c). Results for these 89 variants were incorporated into the VarCall algorithm in a joint analysis with the data from all variants previously published to predict the likelihood of pathogenicity.^6^

### VarCall predictions of pathogenicity

The data analysed here corresponds to a joint analysis of 250 individual *BRCA1* missense variants and a total of 3,695 data points (Supplementary Table S3). The output from VarCall represents the likelihood of pathogenicity given the effects on the functional capacity of the variant. The activity of each variant is represented by a boxplot summarising the marginal posterior distribution of its random effect (Figure 1d; Supplementary Table S4). The landscape of the point estimates of the mixture model is shown in Figure 1d. Note that due to the large size of the figure, variant labels have been omitted to allow focus on the general landscape of the distribution. A detailed summary of the VarCall analysis and variant-specific effects can be found in Supplementary Table S4 and Supplementary Figure S1. The top component of the point estimate corresponds to wild-type control and variants with no impact on TA, and the bottom component corresponds to variants, such as the M1775R negative control, with impaired TA. The present joint analysis contains 214 *BRCA1* variants not previously classified by the multifactorial model^10,11^ as Class 1–2 (not pathogenic or likely not pathogenic) or Class 4–5 (likely pathogenic or definitely pathogenic).

Following the classification scheme proposed by Plon *et al.* ^12^ that summarises the posterior probability in favour of a variant’s pathogenicity on a scale of 1 to 5 with specific probability thresholds, we propose using the posterior probability calculation of a variant being pathogenic in the TA assays (PrDel) output by VarCall to generate a functional classification (fClass) scheme that would classify PrDel<0.001 as fClass 1 (non-pathogenic), 0.001<PrDel⩽0.05 as fClass 2 (likely not pathogenic), 0.05<PrDel⩽0.95 as fClass 3 (uncertain), 0.95<PrDel⩽0.99 as fClass 4 (likely pathogenic), and PrDel>0.99 as fClass 5 (pathogenic). Using the fClass-scoring scheme, only 27 variants remain assigned as VUS (fClass 3). The remaining *BRCA1* missense variants would be classified as either pathogenic (52 variants; fClass 4 and 5) or non-pathogenic (135 variants; fClass 1 and 2; Figure 1d; Supplementary Table S4).

Six of the 89 new variants without previous annotation (M1652K, T1691K, C1697Y, G1748D, C1787S/G1788D and A1789T) significantly impair BRCA1 protein TA function and would be classified as pathogenic (fClass 5). The majority of the variants have a functional classification of non-pathogenic (79 variants; fClass 1 and 2), with only four variants falling in the uncertain category (L1404P, F1571S, R1699P, and H1746Y; fClass 3; Supplementary Table S4). Two of the variants chosen for retest analysis were the C1787S and G1788D. The C1787S variant previously scored as non-pathogenic,^13^ but was classified as IARC 5 using the genetic data.^14^ As it was always seen in conjunction with G1788D (likely in *cis*),^14^ we tested these variants together and separately. Separately, neither variation had a significant impact on protein function in the TA assay, but their presence in the same construct significantly impaired TA levels of the BRCA1 protein (Supplementary Figure S1). We also confirmed that V1833M is a variant with intermediate activity in fClass 4 (Supplementary Figure S1 and Supplementary Table S4).

We further explored the region based on the arrangement of secondary structures in the BRCA1 protein, which was partitioned into 34 segments (coiled-coil, α- helices, β-sheets and intervening segments), 23 of which had at least three variants tested. Some segments are extremely tolerant to changes such as the disordered (82 variants tested) and BRCT α1 regions (7 variants tested), which had no variant in fClass 4 or 5. Conversely, segments in the linker regions Lβ1 and Lα2 are extremely sensitive to aa changes where 6/6 and 8/10 of the variants tested are pathogenic (fClass 4 and 5; Supplementary Table S5).

In the extended construct (aa 1,315–1,863), all of the 10 variants tested in the N-terminus of the coiled-coil domain or the preceding segment were fClass 2 (Figure 1d; Supplementary Table S4), including T1394I predicted by align GVGD to be deleterious (i.e., score C65; Supplementary Figure S2). This suggests that the region is unlikely to contain variants that impact BRCA1 protein function.

### VarCall performance

**Figure 2:**
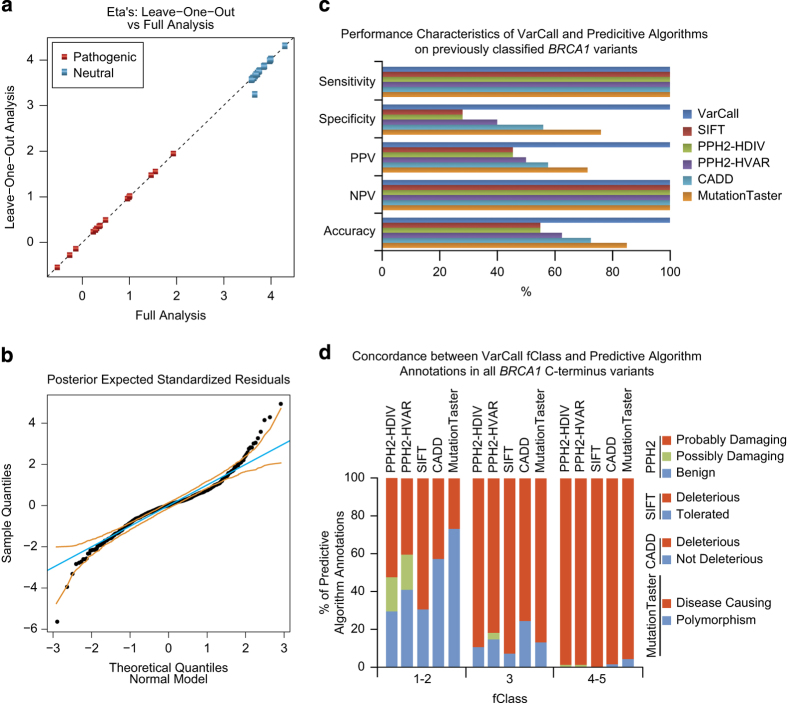
Evaluation of the VarCall model. (**a**) Leave-one-out versus full analysis for each of the 40 known pathogenic or non-pathogenic *BRCA1* variants using VarCall. (**b**) Normal QQ plot of standardised residuals from the fit of the functional assay data were used to model the goodness of fit for VarCall. Standardised residuals were averaged over posterior uncertainty in the model’s parameters. The orange lines mark the simultaneous 95% interval estimate under the normal model. (**c**) Performance characteristics of VarCall compared with SIFT, PolyPhen-2 (HDIV and HVAR), CADD and MutationTaster on the same data set used in **a**. Estimates for sensitivity, specificity, PPV, NPV and accuracy were calculated for each method. (**d**) The concordance of VarCall fClass designations was determined for each of the other five predictive algorithms by comparing overlapping classification terms.

The performance of the VarCall model was assessed using a leave-one-out cross-validation exercise, where in any given run of the model only one of the known variants is left unlabelled. Using a reference panel of 40 known variants classified by multifactorial models^14–16^ (Supplementary Table S6) the assay displayed 1.0 sensitivity (lower bound of 95% confidence interval=0.75) and 1.0 specificity (lower bound of 95% confidence interval=0.83). This analysis achieved good separation of the known neutral and pathogenic variants in the plots of the ‘eta’ values in the leave-one-out versus full analysis (Figure 2a), indicating that the model can be used to classify VUS reliably. A quantile–quantile (QQ) plot of standardised residuals from the final VarCall model, averaged over the posterior parameter uncertainty was generated (Figure 2b). A simultaneous 95% interval estimate for the empirical quantiles includes the observed quantiles. This indicates that the error structure of the model accurately describes residual variability in the data. Therefore, the VarCall model has excellent performance characteristics that accurately classify *BRCA1* variants based on TA assays.

In addition, the performance of VarCall was compared with a sample set of predictive tools commonly used for variant annotation including SIFT,^17^ PolyPhen-2^18^, CADD,^19^ and MutationTaster2^20^. Importantly, this comparison is for general reference only and is not meant as a direct performance comparison because these tools differ in design and objectives (see Discussion). The reference panel of 40 known *BRCA1* variants classified by multifactorial models^14–16^ (Supplementary Table S6) was used to query the functional annotation software tool ANNOVAR,^21^ which provides prediction scores for SIFT, PolyPhen-2 (HDIV and HVAR), CADD and MutationTaster2 (Supplementary Table S7). These results were then used to calculate sensitivity, specificity, negative predictive value, positive predictive value and accuracy for each of these programs (Figure 2c). As mentioned above, VarCall exhibits 100% sensitivity and specificity on the pre-classified BRCA1 variants 100% PPV, NPV and accuracy estimates. The other predictive algorithms also exhibit 100% sensitivity on this data set, but their specificity estimates are relatively poor, ranging from 28–76%, compared with VarCall. Not surprisingly, these results show that a tool to predict pathogenicity using direct functional measurements is superior to exclusively *in silico* predictions.

We also analysed the concordance between VarCall fClass results for all VUS tested in this study and each of the other predictive tools (Figure 2d; Supplementary Table S8). There is a high correlation in fClass 4–5 (likely pathogenic and pathogenic) designated as damaging/deleterious/disease causing by the other methodologies. Most fClass 3 (uncertain) variants have a tendency toward being called damaging/deleterious/disease causing rather than benign/tolerated/not deleterious/polymorphism and the highest degree of discrepancy between VarCall and the other tools is in the fClass 1–2 (non-pathogenic and likely non-pathogenic).

### BRCA1–PALB2 interactions affect transcriptional activation

The impact on transcriptional activation associated with BRCA1 protein variants at aa positions outside of the BRCT domain is limited (Figure 1d). However, several of the variants in the CC domain fell into the fClass 3 (uncertain), including L1404P, L1407P and M1411T that displayed a significant reduction in the levels of TA (Supplementary Figure S1). L1407P and M1411T scored towards the upper limit of this category (PrDel=0.865 and 0.923, respectively) suggesting an increased probability of being pathogenic, whereas L1404P scored lower in the fClass 3 category (PrDel=0.137; Supplementary Table S4). These results suggest that genetic variation in the CC domain affect TA by the BRCA1 protein, but to a lesser extent than those in the BRCT domain.

**Figure 3:**
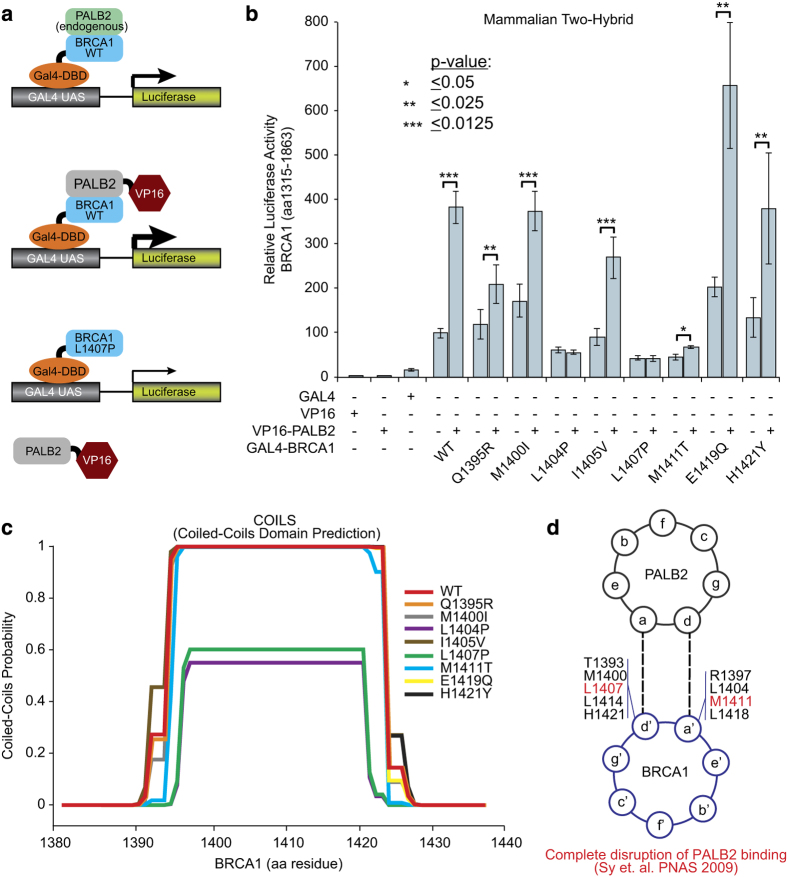
BRCA1 CC variants disrupt PALB2 interaction. (**a**) Schematic of the mammalian two-hybrid assay used to determine BRCA1–PALB2 protein–protein interaction. (**b**) Mammalian two-hybrid results for *BRCA1* CC variants and controls. Variants that disrupt the BRCA1–PALB2 protein–protein interaction lack activation when expressed with the *PALB2*-VP16 construct. (**c**) Probability of CC domain formation in *BRCA1* variants using COILS domain prediction algorithm. (**d**) Schematic of the CC domains of the BRCA1 and PALB2 proteins and the positions that mediate the interaction.

To further explore the CC domain variants, we examined the protein–protein interaction between BRCA1 CC variants and PALB2 using a mammalian two-hybrid assay. Carriers of loss of function variants in *PALB2* also are associated with high risk of breast cancer.^22^ In this assay, wild-type BRCA1 protein bound to the VP16–PALB2 fusion protein enhances the transcriptional activation, whereas BRCA1 CC-containing variants that disrupt the interaction with PALB2 fail to exhibit this transcription enhancement (Figure 3a). Eight *BRCA1* variant constructs (Q1395R, M1400I, L1404P, I1405V, L1407P, M1411T, E1419Q and H1421Y) were tested in this system. The BRCA1 wild-type protein demonstrates a 3.8-fold increase in luciferase activity in this system when co-expressed with the VP16–PALB2 fusion protein, indicating a stable interaction between these two proteins (Figure 3b). Variants L1404P and L1407P were significantly refractory to VP16–PALB2 transcriptional enhancement (Figure 3b). Although M1411T exhibited a statistically significant increase in activity with the addition of VP16–PALB2, the total level of transcriptional activation was still below basal wild-type levels (Figure 3b), consistent with previous experiments (Supplementary Figure S1). L1404P and L1407P are predicted to disrupt the formation of the CC domain structure using the COILS prediction algorithm^23^ (Figure 3c), and both M1411T and L1407P completely disrupt BRCA1–PALB2 protein binding (Figure 3d).^24^ Regardless of the mechanism of disruption, mutations that impair the BRCA1–PALB2 protein–protein interaction are likely to have significant clinical implications.

### Verifying annotation of germline variants by structural inference

The joint analysis described here provides the basis to assess the extent to which we can use variant annotation in one protein domain (i.e., BRCT domains of the BRCA1 protein) to annotate variants in other genes coding for proteins containing BRCT domains that are critical for the cellular response to DNA damage^25,26^ with implications for cancer predisposition (e.g., *BRCA1, BARD1* and *NBN*) and therapy (e.g., *PARP1*).

**Figure 4:**
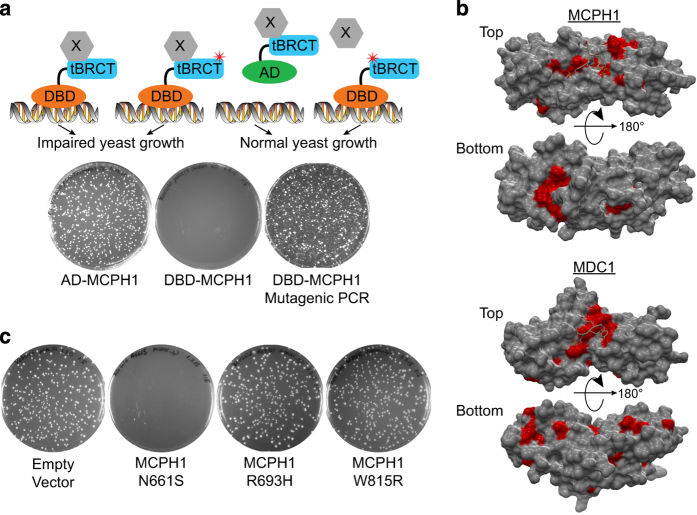
Mutagenesis screen to identify loss of function missense variants in MCPH1 and MDC1 tandem BRCT domains. (**a**) Expression in yeast of proteins containing the tandem BRCT of MCPH1 or MDC1 fused to the GAL4 DNA-binding domain (DBD), but not to the activation domain (AD) of GAL4, lead to growth inhibition and a small colony phenotype. Libraries of mutagenised constructs coding for the tandem BRCTs of *MCPH1* or *MDC1* were transformed into yeast and transformants with regular size colonies (carrying loss of function mutants) were isolated and sequenced to identify residues that disrupt the BRCT function. (**b**) Loss of function variants (red) cluster around the BRCT phosphopeptide-binding pocket in MCPH1 (PDB ID: 3U3Z) and MDC1 (PDB ID: 2AZM). (**c**) *MCPH1* BRCT variants found in cancer samples and predicted to impair function, as inferred by *BRCA1* data sets, (R693H and W815R) abrogate the small-colony phenotype, whereas a predicted neutral mutation (N661S) does not.

Some BRCT-containing proteins when expressed as a fusion that tethers them to DNA induce a DNA damage response in the absence of DNA damage in a function that requires the intact BRCTs.^25,27^ When expressed in yeast as a fusion to the GAL4 DNA-binding domain, but not as a fusion to the GAL4 activation domain (AD), the tandem BRCT of the MCPH1 and MDC1 proteins lead to a small colony phenotype (Figure 4a). We therefore used this phenotype as a functional readout to test the tandem BRCTs of MCPH1 and MDC1 subjected to error-prone mutagenesis. Libraries of mutagenised constructs were transformed into yeast and the large normal phenotype colonies (carrying mutants that disrupt the BRCT domains) were isolated and sequenced to identify residues that disrupt the BRCT function (Figure 4a).

Screening for mutations in BRCT-coding regions revealed 30 and 34 unique missense variants in *MCPH1* and *MDC1,* respectively, which restore normal growth (Supplementary Table S9). Ten and eight recurring missense variants were found in *MCPH1* and *MDC1*, respectively, which indicate residues essential to normal protein function and a strong negative selection in these assays. The 64 unique missense variants were further annotated by alignment with the BRCA1 tBRCT aa sequence (Supplementary Table S9). Thirty-two variants are in aa residues whose equivalent position in the BRCA1 protein has either been classified as IARC4/5 or fClass4/5 (this study) corroborating its functional impact on BRCT structure. Seven additional variants are in aa residues equivalent in the MCPH1 and MDC1 proteins found in both screens and four affect known residues involved in phosphopeptide-binding pocket or salt bridge formation (Supplementary Table S9). Similar to *BRCA1* BRCT pathogenic variants, recurrent variants cluster on the phosphopeptide-binding pocket highlighting the importance of this function (Figure 4b). In addition, this assay identifies other sites on the BRCT domain structure that are important for its normal function and suggest the presence of additional essential protein interaction surfaces.

### Verifying annotation of somatic (tumour) variants by structural inference

To determine whether structural inference could also be used to annotate somatic *MCPH1* variants, we identified 18 variants documented in COSMIC or TCGA and compared them with known *BRCA1* functional variants (Supplementary Table S10). On the basis of this annotation, two variants (R693H and W815R) predicted to have a strong functional impact and one variant (N661S) predicted to have no functional impact were chosen. These variants were then generated by site-directed mutagenesis in the *MCPH1* construct and expressed in yeast (Figure 4c). As expected, the R693H and W815R variants abrogated the small colony phenotype, whereas the N661S variant showed no functional impact (Figure 4c).

Thus, this study identifies functional elements in MCPH1 and MDC1 BRCT domains potentially involved in cell cycle regulation and shows that they correspond to equivalent residues in the BRCA1 BRCT domains. Taken together these results suggest that variants in BRCT domains of non-*BRCA1* genes can be functionally inferred using comparative alignments with the BRCA1 protein BRCT domains and the extensive functional annotation available therein.

## Discussion

The effective use of genomic data to inform clinical decisions is predicated on high-quality annotation of variants as to their likelihood of pathogenicity. Thus, VUS pose a significant hurdle in the use of genetic testing data to improve outcomes. The very low minor-allele frequency of individual variants makes family and population-based approaches difficult to conduct. Thus, in order to provide variant annotation other methods such as functional assays should be used.

Here we hypothesised that functional assays could reliably annotate variants according to their likelihood of pathogenicity for clinical use. To test this hypothesis we experimentally assessed over 100 additional *BRCA1* germline missense variants and conducted a joint analysis of over 250 variants. This data set that represents all documented missense variants located in the C-terminus BRCA1 protein (aa 1,396–1,863), was used to perform validation and determine the likelihood of pathogenicity using the VarCall computational model. This analysis allowed us to assess the clinical relevance of a large number of variants and showed that incorporating the functional data into clinical classifications of *BRCA1* variants would greatly decrease the number of non-informative test results. Applied to the current set of *BRCA1* variants, use of the functional data and VarCall would significantly reduce the number of VUS associated with BRCA1 tested in this study by ~87%.

The VarCall analysis provides a more granular view of segments important for function and revealed secondary structures in the BRCT domains that are unexpectedly tolerant to missense alterations. As all BRCT variants are currently assigned an integrated prior probability higher on average than variants outside key RING and BRCT domains for the purposes of the multifactorial model,^11,28^ the data can guide further calibration of prior probability estimates.

VarCall differs in an important way from other commonly used tools to aid in the annotation of variants such as SIFT,^17^ PolyPhen-2,^18^ CADD^19^ and MutationTaster2.^20^ VarCall uses direct functional measurements to predict pathogenicity while SIFT and Polyphen-2 use multiple sequence alignments to predict the damaging effects of missense variants on protein function; and CADD and MutationTaster2 integrate diverse annotation data (including SIFT and Polyphen scores in CADD) to predict pathogenicity. Thus, our assessment of performance characteristics using the *BRCA1* data set is not meant as a direct comparison but as reference to highlight how different tools can be used in a complementary manner to accelerate variant annotation. VarCall achieves a strong performance in predicting pathogenicity but relies primarily on large data sets collected from detailed functional analysis. Large-scale sequencing projects have identified an extremely large number of germline and somatic genetic variants in humans across Mendelian disorders,^29^ complex traits^30^ and cancer^31^ and most have no prior functional annotation. Moreover, many map to uncharacterised genes. *In silico* tools present a clear advantage for overall annotation because they do not require detailed functional data but their performance may still be insufficient to annotate variants for clinical use. The results presented here indicate that incorporating functional measurements into models designed to distinguish pathogenic from non-pathogenic variants has the potential to enhance our ability to annotate variants in a manner that can be used for clinical decisions.

The present analysis also allowed us to explore variants with intermediate effects. We identified variants in the CC domain with intermediate effects in transcription that correlate with failure to interact with the PALB2 protein (Figure 3). The BRCA1 CC domain interaction with PALB2 is important for cellular response to DNA damage,^32^ and *BRCA1* variants found in cancer patients that disrupt the interaction with the PALB2 protein exhibit defective homologous recombination repair.^24^ Although it is unclear the extent to which a variant with intermediate effects in a functional assay reflects cancer risk, our analysis suggests that even small differences observed in the TA assay are potentially significant.

Caution is warranted when interpreting results from an assay focusing on a single specific biochemical activity to predict pathogenicity. Currently, both the sensitivity and specificity estimates are based on a small number of clearly pathogenic or non-pathogenic variants. Also, variants may affect biochemical functions that contribute to cancer susceptibility but are not being interrogated by the assay. This is a significant challenge for proteins with multiple biochemical and biological functions such as BRCA1. The excellent correlation between results of the transcriptional assay and other biochemical (proteolysis and phosphopeptide binding activity)^33^ or biological (functionally complement BRCA1-deficient mouse embryonic stem cells)^34^ assays indicates that the assay is a sensitive monitor of structure integrity of the BRCT domains. However, it is unclear the extent to which this principle also applies to other regions of the protein or to intermediate variants with intermediate effects. Therefore, discriminating a true intermediate function variant from a neutral or fully pathogenic variant remains difficult and integration of multiple functional assays may be necessary.

Mapping *BRCA1* pathogenic variants to the BRCT 3-dimensional structure highlights the importance of aa residues implicated in phosphopeptide recognition and provides a strong molecular link between this biochemical function of the BRCA1 protein and cancer predisposition. This is also supported by the clustering of loss of function variants in the BRCT domains of the MCPH1 and MDC1 proteins around the phosphopeptide-binding pocket.

In summary, using an extensive functional analysis of *BRCA1* variants mapping to the C-terminal domain of the protein we show that functional assays are robust tools to clinically annotate variants even in the absence of additional data, in the sense that their accuracy matches or surpasses current medical tests. Further, a preliminary analysis of missense variants in *MCPH1* and *MDC1* suggests structural inference may help reliably annotate variants in modular domains found in multiple proteins. Importantly, although functional assays can correctly classify variants and lead, for example, to the reassessment of genetic data to identify the hypomorphic *BRCA1* variant V1713A,^35^ nucleotide changes may have additional effects in splicing or stability not interrogated by the assay, and effects on these processes should be evaluated before a conclusion can be made about functional impact.

The work described here builds on a large body of work on functional assays on cancer predisposition genes such as *TP53*, *BRCA1*, *BRCA2* and *MSH2* ^36–40^ and supports the notion that despite limitations the use of functional assay data is likely to contribute to assessment of an increasingly larger share of VUS and provide more accurate integrated risk models to achieve better clinical outcomes.

## Materials and methods

### Plasmid constructs

The human reference *BRCA1* cDNA region coding for aa residues 1396–1863 (GenBank accession U14680) was cloned into pCDNA3 (ThermoFisher Scientific, Waltham, MA, USA) as a fusion to the GAL4 CAN-binding domain (DBD) domain, as previously described.^9^ Site-directed mutagenesis was performed with the indicated primer pairs (Supplementary Table S11) using the QuickChange II XL kit (Agilent, Santa Clara, CA, USA). Sanger sequencing confirmed all mutations. For the BRCA1 protein aa 1,315–1,863 expression constructs, site-directed mutagenesis was performed using the pcBRCA1-385 (gift from Michael Erdos) plasmid as template with the indicated primer sets (Supplementary Table S11). Amplified products were digested with *Eco*RI and *Bam*HI and cloned downstream of the *GAL4* DBD in the pGBT9 plasmid to create the fusion constructs. The *GAL4* DBD-*BRCA1* segment was excised using *Hind*III/*Bam*HI digestion then subcloned into the pCDNA3 mammalian expression vector. The *VP16 AD*–*PALB2* fusion construct was generated by amplification of *PALB2* (aa 1–319) from normal human leukocyte cDNA using the indicated primers (Supplementary Table S11). The *Eco*RI/*Bam*HI digested fragment was cloned downstream of the *VP16* AD in the pVP16 mammalian expression vector (Clontech, Mountain View, CA, USA).

### Choice of variants

We retrieved all 84 *BRCA1* missense variants deposited the BIC database (http://research.nhgri.nih.gov/bic/) that had not been analysed in the TA assay (Supplementary Table S2). In addition, we tested five novel variants: S1486C (HGVS c.4456A>T), S1580Y (HGVS c.4739C>A), C1697Y (HGVS c.5090G>A), H1746Y (c.5236C>T) and L1844P (HGVS c.5531T>C). We also retested nine variants N1647K (HGVS c.4941C>A), V1696L (HGVS c.5086G>C), G1706E (HGVS c.5117G>A), M1783T (HGVS c.5348T>C), G1788D (HGVS c.5363G>A), A1823T (HGVS c.5467G>A), L1844P (HGVS c.5531T>C), V1833M (HGVS c.5497G>A) and C1787S (HGVS c.5360G>C) that had displayed variable results in previous tests.

To explore the region preceding the CC domain we chose ten variants, three of which represent variants found to date in the population (E1352K, C1372Y and Q1395R) and the remaining seven were included to represent a range of Align GV/GD^41^ scores from C0 to C65 (Supplementary Figure S2).

To probe the CC domain we chose variants located in select residues in region aa 1,392–1,424 mediating the BRCA1–PALB2 protein–protein interaction (Figure 3) and predicted to disrupt (L1404P, L1407) or not (Q1395R, M1400I, I1405V, M1411T, E1419Q and H1421Y) coiled-coil formation by PAIRCOIL2 program using *P*-value cutoff of 0.01 per residue.

### Transcriptional assays and VarCall

All new and retested variants were analysed using the TA luciferase reporter assay as previously described.^13^ Briefly, *BRCA1* constructs were co-transfected in HEK293FT cells with the pG5Luc plasmid, encoding a Luciferase reporter gene driven by GAL4 binding sites, and the phGR-TK plasmid, which constitutively expresses the internal control *Renilla* luciferase. Transcriptional activity was assayed with the Dual-Luciferase Reporter Assay System (Promega, Madison, WI, USA) 24 h after transfection. Variants were tested in at least two independent experiments, with three replicates in each experiment, and assay data were analysed using the computation model VarCall.^6^ Briefly, VarCall is a Bayesian hierarchical model for variant function that accounts for batch-to-batch variation and aa context via random and fixed effects, respectively. Variant function effects, denoted ‘eta,’ have a bimodal, two-component mixture model distribution with one component describing variation among neutral and the other describing variation among pathogenic variants; we interpret the probability that a variant’s eta arises from the pathogenic component as the probability of pathogenicity and its eta as a measure of function.

### ANNOVAR functional prediction

The BRCA1 C-terminus variants analysed in this study were also queried against commonly used predictive methods, including SIFT, PolyPhen-2 (HDIV and HVAR), CADD and MutationTaster2 using the ANNOVAR software tool.^21^ The ANNOVAR package was downloaded and installed from http://www.openbioinformatics.org/annovar/annovar_download_form.php. Current databases were downloaded following the website’s Quick Start-Up Guide and the table_annovar.pl program was used to retrieve results for each of the predictive algorithms listed above.

### Mammalian two-hybrid assay

*GAL4 DBD*-*BRCA1* constructs generated for the TA experiments were used as the bait and co-transfected with the pG5Luc and phGR-TK reporter plasmids. The VP16 AD–PALB2 fusion protein acted as the prey protein in this system. The *BRCA1* variant L1407P was used as a negative interaction control.^24^ When the BRCA1–PALB2 protein–protein interaction occurred, the transcriptional activity was enhanced above the levels observed for the *BRCA1* construct alone due to transcriptional activation mediated by the VP16 AD fused to the PALB2 protein.

### MCPH1 and MDC1 yeast functional assays and error-prone mutagenesis screen

Fragments coding for the tandem BRCT domains of MCPH1 (aa 649–832) and MDC1 (aa 1,894–2,079) were obtained by PCR amplification (Supplementary Table S11) and cloned into the pGBKT7 or pGADT7 vectors (Clontech) as fusions to the GAL4 DBD or AD, respectively. pGBKT7 BRCT, pGADT7 BRCT or empty pGBKT7 were transformed in the Y2HGold *Saccharomyces cerevisiae* strain and plated on dropout medium lacking Tryptophan (SD-Trp) or Leucine (-Leu) and number of colonies was scored.

For mutagenesis assays, the mutagenised libraries were generated by error-prone PCR using pGBKT7 containing the tandem BRCT domains of MDC1 or MCPH1 as templates. Mutagenesis was performed using *Taq* DNA PCR (initial denaturation: 94° C for 3 min; 60 cycles; 94 °C for 45 s, 63 °C for 30 s, 72 °C for 90 s; final hold at 72 °C for 10 min) using designated primers (Supplementary Table S11).

The PCR product with the correct size was gel purified and co-transformed with an equimolar ratio with the linearised pGBKT7 MDC1 tBRCT or pGBKT7 MCPH1 tBRCT into *Saccharomyces cerevisiae* Y2H Gold. The linearised plasmids were generated by single restriction digest of MDC1 and MCPH1 using *Bgl*II, and *Spe*I, respectively. Cells were plated on SD–Trp plates and revertants (regular size colonies) were isolated and lysed. BRCT regions were amplified by KOD Polymerase PCR (Supplementary Table S11) using Matchmaker Insert Check PCR Mix 2 (Clontech) for mutation identification by Sanger sequencing. Variants were mapped to the 3D structures of MDC1 (PDB ID 2AZM)^42^ and MCPH1 (PDB ID: 3U3Z)^43^ in complex with phosphorylated histone H2AX.
