# Supplementary figures and images for: Functional assays provide a robust tool for the clinical annotation of genetic variants of uncertain significance
Source: NPJ Genom Med. 2016 Mar 2;1:16001–. doi: 10.1038/npjgenmed.2016.1 (PMC5539989; doi:10.1038/npjgenmed.2016.1)

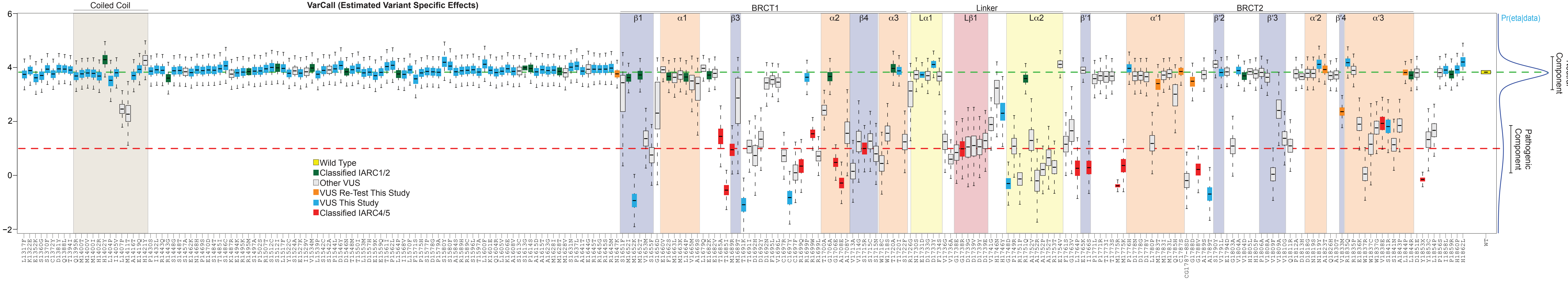

Supplement: Supplementary Figure S1 [file npjgenmed20161-s1.pdf]
