# Supplementary material for: Functional assays provide a robust tool for the clinical annotation of genetic variants of uncertain significance
Source: NPJ Genom Med. 2016 Mar 2;1:16001–. doi: 10.1038/npjgenmed.2016.1 (PMC5539989; doi:10.1038/npjgenmed.2016.1)
Supplement: Supplementary Figure S2 [file npjgenmed20161-s2.pdf]

Figure S2

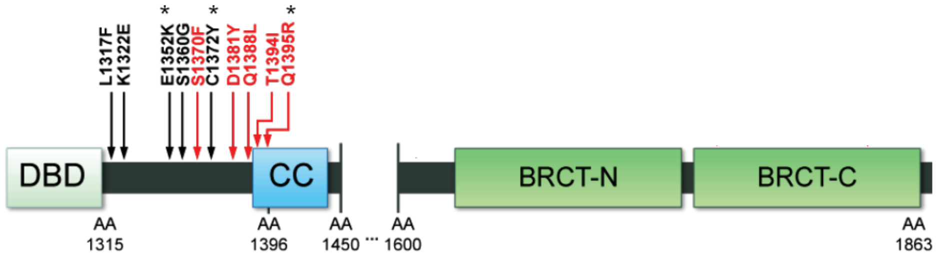

Protein Variant (HGVS)	Exon	DNA Variant (HGVS)	Region	Natural Variant	Context (aa range)	Align GV/GD
p.E1352K	exon11	c.4173G>A	Disordered	Yes	1315-1863	C0
p.C1372Y	exon12	c.4234G>A	Disordered	Yes	1315-1863	C0
p.Q1395R	exon12	c.4303A>G	Coiled-coil	Yes	1315-1863	C35
p.L1317F	exon11	c.4070G>C	Disordered	no	1315-1863	C0
p.K1322E	exon11	c.4083A>G	Disordered	no	1315-1863	C0
p.S1360G	exon11	c.4197A>G	Disordered	no	1315-1863	C0
p.S1370F	exon12	c.4228C>T	Disordered	no	1315-1863	C15
p.D1381Y	exon12	c.4260G>T	Disordered	no	1315-1863	C15
p.Q1388L	exon12	c.4282A>T	Disordered	no	1315-1863	C15
p.T1394I	exon12	c.4300C>T	Coiled-coil	no	1315-1863	C65
