# Supplementary material for: Functional assays provide a robust tool for the clinical annotation of genetic variants of uncertain significance
Source: NPJ Genom Med. 2016 Mar 2;1:16001–. doi: 10.1038/npjgenmed.2016.1 (PMC5539989; doi:10.1038/npjgenmed.2016.1)
Supplement: Supplementary Figures and Tables Legends [file npjgenmed20161-s14.doc]

**Supplementary Figure S1.** VarCall analysis of missense variants in the carboxy-terminal region (aa1315-1863) of the BRCA1 protein; related to Figure 1D containing variant labels.

**Supplementary Figure S2.** Diagram and details of *BRCA1* variants tested in the aa 1315-1863 context.

**Supplementary Table S1. Results from transcription activation assays.** Firefly luciferase activity was normalized by the internal control (*Renilla* luciferase) and reported as percent of wild type BRCA1 protein activity.

**Supplementary Table S2. *BRCA1* variants analyzed by transcriptional activation assays in this study.** All DNA and protein annotations correspond to the HGVS annotation. # indicates variants re-tested in this analysis. Clone 97 is a construct containing both the C1787S and G1788D mutations.

**Supplementary Table S3. VarCall input data.** Raw transcriptional data indexed by batch.

**Supplementary Table S4. VarCall-generated Posterior probabilities of *BRCA1* missense variants and fClass assignments.** PrDel values were used to assign variants fClass scores and categories.

**Supplementary Table S5. Segment analysis of secondary structures in the C-terminus of BRCA1.**

**Supplementary Table S6. Transcriptional Assay Validation panel of variants.** *BRCA1* variants previously classified by the multifactorial model in IARC Classes 1 & 2 (non-pathogenic) and 4 & 5 (pathogenic) reported by Lindor et al. 2012 and Vallée et al. 2012 used to validate the TA and assess the performance characteristics of VarCall and the other predictive methodologies.

**Supplementary Table S7. Analysis of performance characteristics of commonly used predictive tools against pre-classified *BRCA1* variants.** SIFT, PolyPhen-2 (HDIV & HVAR), CADD, and MutationTaster2 programs were queried using the ANNOVAR functional classification software package. Algorithm specific scores and classifications in order to obtain estimates for each method’s sensitivity, specificity, NPV, PPV, and accuracty in Fig. 2C.

**Supplementary Table S8. Analysis of the complete list of *BRCA1* C-terminus variants by other predictive methods.** ANNOVAR was used to retrieve results for SIFT, PolyPhen-2 (HDIV & HVAR), CADD, and MutationTaster2 to compare the concordance with VarCall fClass assignments depicted in Fig. 2D.

**Supplementary Table S9. *MCPH1* and *MDC1* mutations identified in the yeast mutagenesis screen.**

**Supplementary Table S10. Somatic variants of *MCPH1* found in tumors.** Missense variants retrieved from COSMIC and TCGA databases.

**Supplementary Table S11. PCR Primers used in this study.**
